# Development, growth and metabolic effects in stage IV lobster (*Homarus americanus*) following chronic exposure to sediments spiked with commercial formulations of deltamethrin and permethrin

**DOI:** 10.3389/fphys.2023.1151176

**Published:** 2023-05-05

**Authors:** Dounia Daoud, Spencer J. Greenwood, Benjamin de Jourdan, Davide Asnicar, Laura J. Taylor

**Affiliations:** ^1^ EcoNov Inc., Moncton, NB, Canada; ^2^ Homarus Inc., Shediac, NB, Canada; ^3^ Department of Biomedical Sciences, Atlantic Veterinary College, University of Prince Edward Island, Charlottetown, PE, Canada; ^4^ AVC Lobster Science Centre, Atlantic Veterinary College, University of Prince Edward Island, Charlottetown, PE, Canada; ^5^ Huntsman Marine Science Centre, Saint Andrews, NB, Canada

**Keywords:** pesticides, marine invertebrates, pyrethroids, metabolism, American lobster, LC50, abnormalities

## Abstract

Coastal and estuarine ecosystems are environments heavily influenced by natural and anthropogenic activities. Chemicals used for pest control in agriculture and aquaculture may accumulate in natural coastal environments. Pyrethroids are common pesticides that are used on crops as well as applied to aquaculture pens and then may disperse in the surrounding ocean once treatment is complete. This study observed the sublethal effects of two pyrethroids, permethrin and deltamethrin (within commercially available formulations), on post-larval stage IV American lobster (*Homarus americanus*) using growth parameters and metabolic rate as indicators. Observed effects on growth parameters were a decrease in size increment and specific growth rate as well as an increase in intermolt period in stage IV lobsters exposed to 100 μg/kg permethrin. No significant differences were found for intermolt period, size increment, or specific growth rate in deltamethrin-exposed stage IV lobsters. Metabolic rates were not significantly different between deltamethrin-exposed and control lobsters, however, this sublethal effect warrants further investigation. Collectively, these results represent the first examination of the sublethal effects of exposure to pyrethroids formulations in post-larval lobsters, highlighting the potential for effects on non-target marine organisms.

## 1 Introduction

Coastal areas are peculiar transitional environments subjected to natural and anthropogenic pressures such as tides, wave action, urban and industrial settlements, fishery, tourism, agriculture, and aquaculture. As much as 75%–90% of commercial fisheries in North America depend on estuaries and coastal areas for some portion of their life cycle (Lellis-Dibble et al., 2008).

Pyrethroids are one category of pesticides that are increasingly being used in agriculture as they act as strong insecticides at very low concentrations and present low toxicity to mammals (Hartwell, 2011; Rehman et al., 2014). Pyrethroids are used to control lice, fleas, and ticks on companion animals and livestock, as well as to protect agricultural crops and fish, from arthropod pests (Lu et al., 2019). This category of pesticides is also highly toxic to insects and aquatic arthropodic pests (Antwi and Reddy, 2015). The mode of action of pyrethroids on invertebrates is not fully understood. The current thought is that they disrupt axonal transmissions of nerve impulses by altering ion permeability in squid (Narahashi and Anderson, 1967; Wang et al., 1972; Clark and Matsumura, 1982) as well as inhibiting ATPases involved in active transport in mammals (Gray and Soderlund, 1985), causing disruption to osmoregulation and ion movement within cells (Antwi and Reddy, 2015). The consequences of ion balance disruption are various and dependent on chemical characteristics. Pyrethroids are divided into two groups based on the absence (type I; e.g., permethrin) or presence (type II; e.g., deltamethrin) of a cyano moiety at the alpha position (Nasuti et al., 2003), which changes the mode of action of the pyrethroid. It has been shown in different groups of animals (vertebrates, terrestrial and marine invertebrates) that type I pyrethroids cause tremors, hyperexcitation, ataxia, convulsions, paralysis, prostration, or death, while type II pyrethroids can cause oxidative stress, tremors, restlessness, rolling convulsions, and mortality (Lawrence and Casida, 1982; Rehman et al., 2014; Zhou et al., 2019).

Permethrin is a type I pyrethroid found in over 230 products registered for use in Canada and is widely used in agricultural and urban areas surrounded by wetlands for mosquito control (Canadian Council of Ministers of the Environment, 2006; Rehman et al., 2014; Lu et al., 2019). Permethrin has been found around the world in water bodies adjacent to agricultural fields containing cotton, potato, sugar beet, and vineyards (Antwi and Reddy, 2015), and in coastal sediments at concentrations from 0.004 up to 132 μg/kg (Lao et al., 2010; Adeyeye et al., 2021). Sediment sampling found permethrin detectable in California sediments with one study observing between 1.3 and 459 μg/kg permethrin in rivers, creeks, irrigation canals, and tailings ponds (Weston et al., 2004). In European and African coastal waters, permethrin was found at very low concentrations, below 0.5 ng/L (Anderson et al., 2014; Zhang et al., 2016). In contrast, surveys of stream surface waters and sediment in Atlantic Canada have detected permethrin 110 ng/L (Environment Canada, 2011).

Deltamethrin is a type II pyrethroid used for agriculture and aquaculture arthropod pests (Rehman et al., 2014; Lu et al., 2019). It is the active ingredient in the commercial formulation AlphaMax^®^ which has been used as a treatment against sea lice infestations in salmon aquaculture farms. Following treatment, deltamethrin is released into the surrounding water (Burridge et al., 2014; Lyons et al., 2017). The dispersion range of deltamethrin from an aquaculture treatment site has been found to extend to 986 m so the potential for exposure of non-target organisms is relatively high (Page and Burridge, 2014; Lyons et al., 2017). This risk is especially high for lobsters which tend to settle in coastal areas where aquaculture farms are typically located (Burridge et al., 2014). Due to its low water solubility (<2 μg/L) and a log K_OW_ value of 4.6, deltamethrin is not expected to persist in the aqueous phase and is likely to adsorb to particles and accumulate in sediments (Van Geest et al., 2014), where it has been estimated to have a half-life of ∼140 days (Gross et al., 2008; Van Geest et al., 2014). Nonetheless, deltamethrin has been found in surface water across the globe at concentrations ranging from 0.002 to 4 μg/L (Feo et al., 2010; Mahboob et al., 2015). In Canadian freshwater sources it has been detected at concentrations between 0.010 μg/L to 24 μg/L and concentrations found in sediment were observed between 3 and 5 μg/kg (Canadian Council of Ministers of the Environment, 1999).

Pyrethroids and their breakdown products are hydrophobic and have high octanol-water (K_OW_) and organic carbon-water (K_OC_) partition values so they are typically associated with sediments when found in aquatic environments (Maund et al., 2002; Weston et al., 2004; Lao et al., 2010; Smalling et al., 2010). Benthic organisms may be chronically exposed to pyrethroids and other marine sediment-bound pollutants and the long-term effects to these non-target organisms are largely unknown (Barata et al., 2002).

The American lobster, *Homarus americanus*, is a benthic non-target arthropod that is also one of the most valuable commercial fisheries in Canada. Canadian lobster landings have recently reached all-time highs across all lobster fishing areas (LFA) but within LFA 25 and 26A of the Northumberland Strait, SCUBA surveys of the abundance of 1-year-old lobster (young-of-year, YOY) show a decline which could translate to a future decrease in landings (Comeau et al., 2008; Rondeau et al., 2015; Fisheries and Oceans Canada, 2019). The metamorphosis from pelagic stage III larval lobsters to benthic stage IV lobsters is a critical developmental change that coincides with the organisms settling to the benthos. Stage IV lobster settling is most likely to occur in coastal environments due to the appropriate temperature and availability of shelter (Lawton and Lavalli, 1995), and so more at risk of exposure to pesticides used inland.

Lobsters have been used widely as a model organism to test the effect of pollutants, including pesticides. Indeed, the active ingredients of many formulated products may cause alteration of gene expression, behaviour, growth inhibition and mortality (Reebs and Lague, 2000; Ernst et al., 2001; Horst et al., 2007; Hellou et al., 2009; Fairchild et al., 2010; Reebs et al., 2011; Bauer et al., 2013; Daoud et al., 2014; Taylor et al., 2019). Despite the availability of this data, the linkage between pesticide-induced cellular energy dysregulation with whole body energy imbalance, fitness, and production remains to be determined. Sublethal endpoints, such as metabolic scope, are integrative indices of the energy status of an organism that measure the available energy for aerobic activities (e.g., swimming, growth, reproduction, and feeding) (Daoud et al., 2014).

The present study investigates the effects of commercially available formulations containing permethrin and deltamethrin on mortality, growth, and metabolic rates of stage IV American lobsters. Formulations are composed of additional proprietary ingredients which may increase solubility or absorption when used and may change the toxicity of the active ingredient (Pereira et al., 2009; Asnicar et al., 2020). It was, therefore, important to show the effect of the formulations that are commonly used, and that animals in the wild are more likely to be exposed to, rather than the active ingredient alone. Stage IV lobsters exposed to spiked sediment were chosen for this study as this is the first lobster life-stage with benthic activity. To this end, three experiments were conducted: 1) exposure to permethrin to determine molting inhibition; 2) similarly to Experiment 1, lobster were exposed to deltamethrin to determine molting inhibition; 3) assessment of lobster metabolic rate under pesticide exposure condition. It was expected that the tested pyrethroids would impact growth and metabolism in stage IV lobsters with no significant difference in effect between Type I and Type II pyrethroids.

## 2 Materials and methods

### 2.1 Collection and acclimation of lobsters

Ovigerous females of *Homarus americanus* were collected along the East coast of Canada near Miguasha in the Baie des Chaleurs and transported to the Coastal Zones Research Institute located in Shippagan (NB, Canada). Adult lobsters were kept in 800 L tanks in a flow-through seawater system with water pumped from the sea, kept at 11°C, salinity 28–30 ppm, with a flow rate set at 5 L min^−1^. Upon hatching and subsequent release, stage I larvae were collected and transferred to 1200 L tanks filled with 2 μm filtered, UV-treated seawater at 20°C ± 1°C; salinity of 28–30 ppm, water flow of 1 L min^−1^ and a light:dark photoperiod of 16:8. The larvae were communally raised to stage IV and fed a combination of frozen *Artemia* (Hikari, Kyorin Co. Ltd.) and dry brine shrimp flakes (Salt Creek) twice a day (Chiasson et al., 2015).

Stage IV, post-larvae were transported to the Environment Canada Toxicology Laboratory in Moncton (NB, Canada) where they were kept in the same rearing conditions for 7 days in a recirculating system. Seawater was renewed (80%) three times a week until the beginning of the experiments.

### 2.2 Exposures protocol

Sediment was collected from a control site at Callanders Beach in Kouchibouguac National Park, NB, Canada (N 46° 48.502′; W 64° 54.358′) that is presumed uncontaminated (McIver et al., 2015; Roughan et al., 2018). The top layer of sediment (between 5 mm and 10 mm layer) exposed at low tide was collected between the high and low tide marks using stainless steel spatulas that had been previously cleaned with solvents. Macro vegetation, organisms, or shells were removed by operators by hand picking.

Technical grade formulations commercially available containing permethrin or deltamethrin as the active ingredient were used to prepare test solutions. The active ingredient percentage in the formulations was 38.4% and 1%, respectively. Stock solutions were prepared in seawater, by dissolving 0.1 mL of the formulation in 1 L of 0.22 µm filtered seawater.

Sediment was weighed into stainless steel bowl (2,000 g) and the quantity of stock solution needed to reach the target concentration was added.

For Experiments 1 and 2, the following nominal concentrations were obtained: 0, 3, 10, 33, and 100 μg/kg for permethrin (Experiment 1) and 0, 0.05, 0.5, and 5 μg/kg for deltamethrin (Experiment 2). The treatment concentrations used for permethrin were chosen in accordance with prior experiments at the Environment Canada Toxicology Laboratory (unpublished) and were expected to be sublethal.

After thoroughly mixing in a commercial mixer, 50 g of the spiked sediment was added to each 1 L glass exposure vessel that was subsequently filled with seawater (Environment Canada, 1997). The vessels were then placed in a temperature-regulated water bath at 20°C ± 2°C and covered with a plastic lid. An airline was added to each vessel and the sediment was allowed to settle overnight with light aeration. Once dissolved oxygen in the seawater was measured as > 95%, a single stage IV lobster was housed in each vessel (See Table 1 for distribution of organisms). Each lobster was fed 10 mg of frozen brine shrimp and 10 mg of dry fish flakes daily. Observations on survival and molting were recorded daily, as well as temperature. Moreover, dissolved oxygen, salinity, and pH were checked three times a week on two randomly selected replicates per concentration. After 7 days, 80% of seawater and food were removed and renewed in all the jars.

**TABLE 1:** Allocation of available stage IV lobster postlarvae to endpoints for control and exposed sediment spiked with formulated permethrin and deltamethrin, for 14 and 15 days, respectively. Experiments 1 and 2 have the same endpoints.

Experiment number	1	2	3
Compound	Permethrin	Deltamethrin	Deltamethrin
Concentrations tested (µg/kg)	0, 3, 10, 33, 100	0, 0.05, 0.5, 5	0, 0.05
Initial N of lobster postlarvae per group	34	38	56
Endpoints measured and time (days)			
Survival rates	t = 14	t = 15	t = 15
Growth and development	t = 0, t = 14	t = 0, t = 15	t = 0, t = 15
Metabolic rates	no	no	yes on stage VI

To calculate growth increments, initial cephalothorax length (CL_i_) was measured on a group of fifty 7-day-old stage IV lobsters on day 1 of exposure and final length after molt (CL_f_) was measured on remaining lobsters on day 14, using a digital microscope (Vp-Eye ver. 6.0, Aven Inc., USA) with image analysis software (ImageJ, ver. 1.440, National Institute of Health, USA). Moreover, seven lobsters from each group were photographed and observed for morphologic deformities following the classification of Samuelsen et al. (2014).

### 2.3 Metabolic rate analysis under stress condition

Experiment 2 provided data for finding the sublethal range of deltamethrin for stage IV lobsters. Therefore, in the third experiment, Stage IV lobsters were exposed to deltamethrin at the nominal concentration of 0.5 μg/kg. The exposure setup to the spiked sediment was the same as explained above for Experiment 1 and Experiment 2.

Stage IV lobsters exposed to deltamethrin during Experiment 3 were allowed to molt until stage VI in order to achieve consistency in stage of settled juveniles before metabolic testing began. To assess metabolism, intermittent-flow respirometry was used to monitor respiration rates using a 4-channel oxygen measurement system designed to detect changes in dissolved oxygen. Intermittent-flow respirometry typically produces a range of MO_2_ values (oxygen consumption values per individual in mg O_2_ h^−1^) for each individual animal (Steffensen, 1989). MO_2_ can be elevated by stress, digestion and spontaneous activity, but over several days of recording, a lower limit in MO_2_ values becomes apparent and corresponds to SMR. Each channel was connected to a 5 mL closed chamber containing a single lobster, during each measurement period three lobsters were randomly assigned to an individual chamber and monitored simultaneously. One channel was randomly assigned to run empty (seawater only) during each measurement period to determine background oxygen levels. Optodes (Presens, Germany) were affixed to the inside of each chamber and connected to an oximeter (OXY-4, Presens, Germany) with an online temperature compensation device (TEMP-4, Loligo Systems, Denmark) to provide real-time temperature data.

A total of 6 control and 9 exposed stage VI lobster (3 ± 1 days after molt) were tested for their individual metabolic rates. Each lobster’s metabolism was monitored in batches of 3 lobsters for 4–5 days, in order to reach the lowest metabolic rate possible. Each batch trial was terminated as soon as another batch of stage IV lobster became available.

Prior to transfer to one of the chambers, each lobster was stimulated to swim using a magnetic stir bar to provide a current with a gradual increase to current speed. Once a lobster displayed signs of exhaustion, as evidenced by resistance or inability to swim, it was transferred to an oxygen measurement chamber.

The monitoring session for each lobster lasted 4–5 days and consisted of the continuous repetition of the following steps (each cycle lasted 510 s): 1) 350 s chamber flushing with fresh seawater to reach saturated oxygen levels; 2) stop of the flow; 3) waiting 60 s for oxygen level to settle; 4) 100 s of oxygen measurement. See Supplementary Figures S1, S2 for further explanation.

All measurement devices were controlled and monitored by a computer program designed for intermittent-flow respirometry (Autoresp4; Loligo Systems, Denmark). Background oxygen levels were also obtained by running three cycles of monitoring oxygen levels for 700 s in all 4 chambers, before and after each session with lobsters. Nitrogen gas was used to calibrate 0% oxygen for the optodes. Before each monitoring session, the optodes were calibrated to 100% saturation and during measurement oxygen levels never reached less than 90% saturation. Barometric pressure as well as seawater temperature and salinity were measured daily to calculate the solubility of oxygen.

Metabolic measurements were performed following methods described in Daoud et al. (2007). Metabolic rate was measured immediately after the lobsters were stimulated to swim (maximum metabolic rate (MMR) as well as when the lobsters were at rest (the standard metabolic rate (SMR), approximately 24 h after the MMR measurement. Factorial Metabolic scope (FMS) was calculated by dividing the MMR by the SMR.

### 2.4 Calculations and statistical analysis

The intermolt period (IP) usually represents the duration in days between two successive molts. However, in this study, some lobsters molted twice during the 14 days of exposure reaching stage VI. Thus, SI represents general increase in size between all molts performed during the exposure, from stage IV to stage V or VI.

The relative size increment (SI) was calculated:

$$SI=100x\left({CL}_{f}–{CL}_{i}\right)/{CL}_{i}$$ (1)

where CL_i_ and CL_f_ are the pre-molt and post-molt carapace length (CL), respectively.

In addition, a specific molt duration between stage IV and V was calculated (IP_45_) for further comparisons.

Specific growth rate (SGR) was calculated from the CLs for each stage and the IP parameter, and can be used to describe the combined effects of these two components of growth (Ricker, 1975) for all lobsters:

$$SGR=\left({logCL}_{f}–{logCL}_{i}\right)/IP$$ (2)

Mean values of growth were compared between treatments by 1-way ANOVA followed by Dunnett’s test (Sokal and Rohlf, 1995). Graphical examinations of the data and Brown–Forsythe tests were used to examine homogeneity of variance (Brown and Forsythe, 1974). The values were assessed for normality using the Shapiro-Wilk test. All calculations and statistics were conducted using R 2.14.0 (R Core Team, 2011), and raw data is available in the Supplementary Material and online at FigShare (https://doi.org/10.6084/m9.figshare.20199098.v1).

Time-to-event analysis was performed in order to compare the restricted means of the time it took 50% of the lobsters in each treatment to molt (Schober and Vetter, 2018). In addition, the median effective concentration (EC50) was calculated for lobsters that molted twice during the study.

## 3 Results

### 3.1 Effects of sublethal exposure to permethrin

**FIGURE 1:**
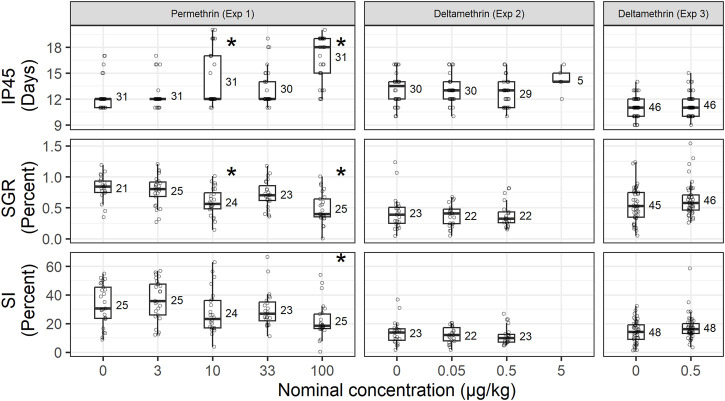
Growth parameters of stage IV lobsters (*Homarus americanus*) exposed to permethrin and deltamethrin formulations at different concentrations. Intermolt period (IP_45_), specific growth rate (SGR), and overall size increment (SI), are shown with the number of observations (n) next to each boxplot. Significant differences (*p* < 0.05) from the control of a given experiment are marked with an *.

**FIGURE 2:**
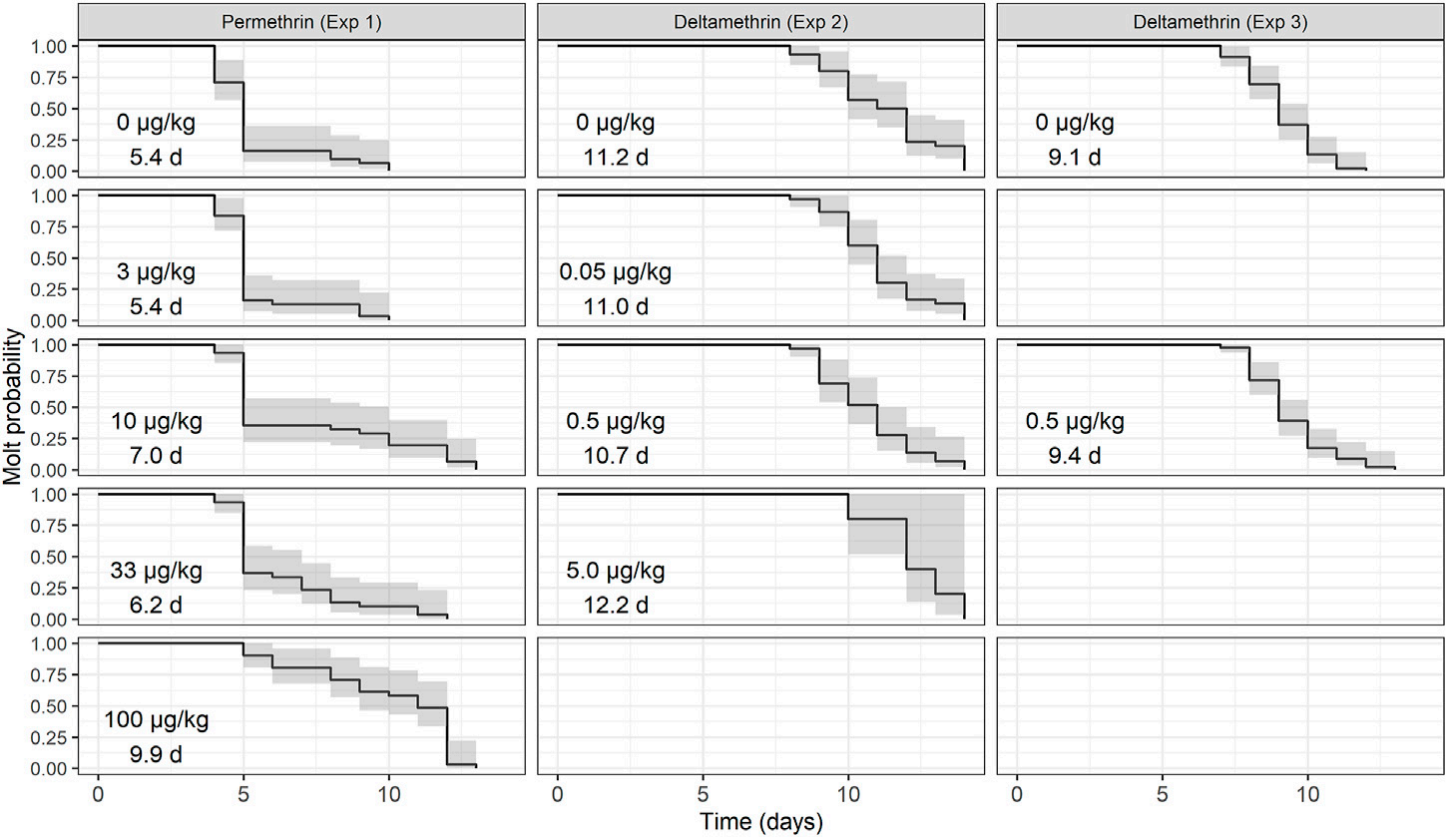
Time-to-event analysis to determine the time at which lobsters, exposed to permethrin and deltamethrin formulations, molt. Each panel shows the nominal concentration and the time to mean molt. The grey areas represent the 95% confidence bands of the Kaplan-Meier curve.

During the exposures to the permethrin formulation (Experiment 1), lobsters in all treatment concentrations survived until the end of the exposure period (14 days) except two lobsters that died in the 33 μg/kg group, confirming that the treatments were sublethal doses. Lobsters were 7 days old on day 1 and had an average CL_i_ of 3.90 ± 0.24 mm. All stage IV post-larvae exposed to permethrin molted to stage V during the experiment. However, there was a difference in the intermolt period (IP_45_, Figure 1) with the 10 and 100 μg/kg treatment taking significantly longer to complete the molt. Time-to-event analysis for molting determined restricted-mean times of 5.4, 5.4, 7.0, 6.2, and 9.9 days from entering the study for 50% of stage IV lobsters to molt to stage V when exposed to 0, 3, 10, 33 and 100 μg/kg respectively (Figure 2).

**FIGURE 3:**
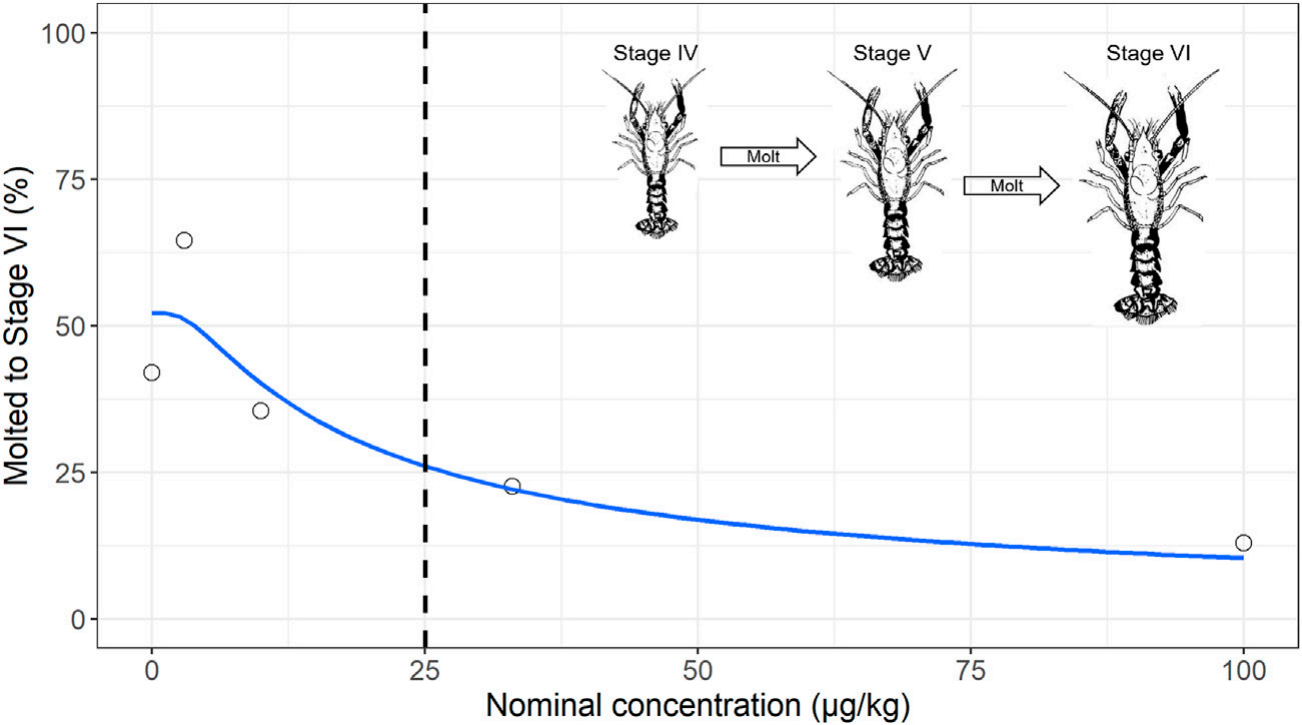
Concentration response curve showing the percent of lobsters which molted to stage VI in the permethrin formulation exposure. The dashed vertical line is the EC50 of 25.1 μg/kg (standard error = 17.9). Insets show that between stages IV to VI, juvenile lobsters undergo changes in size and not in shape or appendages modifications.

The longer time between molts in the higher treatments is further evident in the total number of lobsters that molted twice during the study, with 42, 65, 35, 23% and 13% molting to stage VI in the 0, 3, 10, 33 and 100 μg/kg treatments respectively (Figure 3). The percentage of lobsters molting to stage VI was modelled with a three-parameter type 2 Weibull model and yielded an EC50 of 25.1 μg/kg (standard error = 17.9).

There was a significant difference (F_(4,117)_ = 4.101, *p* = 0.004) in the overall size increase (SI, Figure 1) during the experiment with the 100 μg/kg group having a significantly lower SI (22%) compared to the control (36%) (Dunnett’s *post hoc* test, *p* = 0.008). The decreased SI and increased IP_45_ in the higher permethrin treatments also are reflected in the significant decrease in specific growth rate (F_(4,113)_ = 8.831, *p* < 0.001) for lobsters from the 10 and 100 μg/kg treatments (Dunnett’s *post hoc* test, *p* = 0.004 and *p* < 0.001, respectively), however the SGR for the 33 μg/kg treatment lobsters was not significantly different (Dunnett’s *post hoc* test, *p* = 0.350) from the control (Figure 1).

**FIGURE 4:**
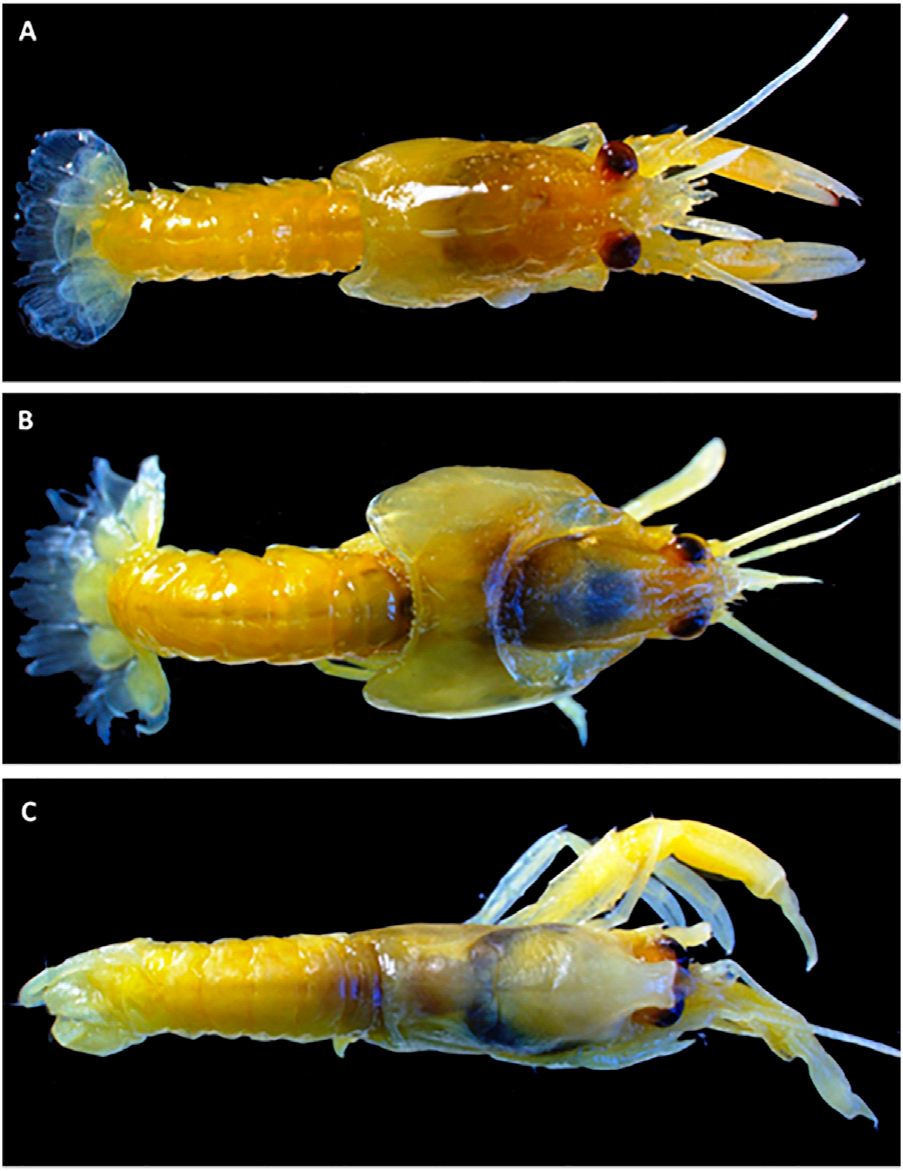
General views of some representative deformations of juvenile lobsters after sediment-bound pesticide formulations exposures. **(A)** Control lobster, **(B)** Permethrin at 33 μg/kg of sediment and **(C)** Deltamethrin at 0.05 μg/kg of sediment. In figures **(B,C)**, lobsters show lack of limbs and antenna, changes in eyes position, as well as malformations in the cephalothorax and abdomen areas, including the tail.

Observations on whole organisms showed severe malformations in lobsters exposed to the permethrin formulation when compared to control lobsters (Figure 4). These malformations included carapace deformities such as bilateral or unilateral separation from the main body, deformities of the setae on the uropods to an elongated and ragged appearance, and claws were missing or were atrophied. Of the seven lobsters observed per group, none presented malformations in the control group, three lobsters (43%) presented deformities in each the 3, 10, and 33 μg/kg groups and five lobsters (71%) were deformed in the 100 μg/kg group.

### 3.2 Effects of sublethal exposure to deltamethrin

In the first experiment with deltamethrin formulation (Experiment 2), 5.3% of lobsters in the control group, 7.9% of those exposed to 0.05 μg/kg, 10.5% of those exposed to 0.5 μg/kg, and 71.0% of those exposed to 5 μg/kg died within the 14-day exposure period, yielding an LC50 of 2.5 μg/kg (standard error = 0.56).

Lobsters sampled at day 1 of deltamethrin exposure had a mean CL_i_ of 4.15 ± 0.21 mm (*n* = 27). No significant differences were found for IP_45_, SGR, or SI in lobster exposed to increasing concentration of deltamethrin (Figure 1). The time to molt analysis determined that it would take 11.2, 11.0, 10.7, and 12.2 days for 50% of exposed lobsters to molt at 0, 0.05, 0.5, and 5 μg/kg respectively (Figure 2).

In the second exposure of benthic stage lobster to deltamethrin formulation (Experiment 3), it was found that it would take 9.1 and 9.4 days for molt to occur in 50% of lobsters exposed to 0 and 0.05 μg/kg respectively (Figure 2). Also, in this case, no significant differences were found for IP_45_, SGR, or SI (Figure 1).

**FIGURE 5:**
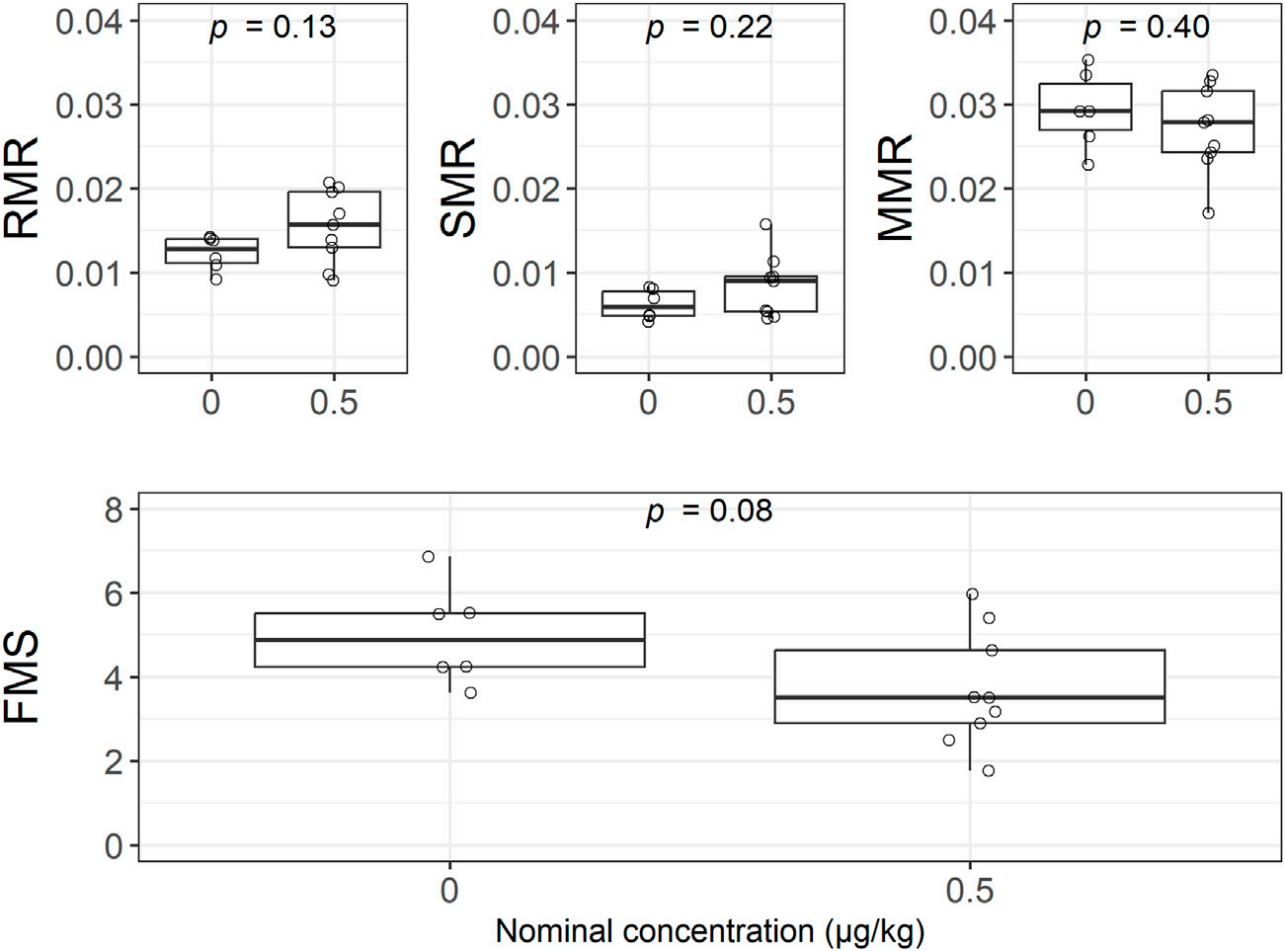
There were no significant differences between the metabolic measures of resting metabolic rate (RMR), standard metabolic rate (SMR), maximum metabolic rate (MMR) or the factorial metabolic scope (FMS) between the control (*n* = 6) and the 0.5 μg/kg deltamethrin formulation treatment (*n* = 9), using the two-tailed t-test.

The various metabolic rate endpoints measured in lobsters exposed to 0.5 μg/kg deltamethrin were not significantly affected when compared to the control (Figure 5). The resting metabolic rate (RMR) was 0.0123 ± 0.002 in the control lobsters and 0.0154 ± 0.004 in the deltamethrin exposed lobsters. Similarly, the standard metabolic rate (SMR) was slightly greater in the exposed lobsters (0.0083 ± 0.004) than in the control group (0.0062 ± 0.002). The maximum metabolic rate (MMR) was nearly equal in each treatment (0.029 ± 0.005 in the control and 0.027 ± 0.005 in the exposed). Lastly, the factorial metabolic scope (FMS) was calculated to be 5.00 ± 1.18 for control lobsters and 3.71 ± 1.37 in group exposed to 0.5 μg/kg deltamethrin.

## 4 Discussion

Since the last century, increasing concerns about the toxic effects of pesticides on non-target aquatic organisms have emerged (Ernst et al., 2001). Knowledge of the ability of invertebrates to deal with anthropogenic environmental stressors is increasing, but gaps are still present. Toxicological assessment of pollutants in lobsters came into the spotlight two decades ago after high mortalities in Long Island Sound had severe repercussions on the local community (De Guise et al., 2005; Walker et al., 2005; Zulkosky et al., 2005; Seara et al., 2022). Following the discharge of pesticides, benthic life forms, and in particular cryptic organisms such as juvenile lobsters (Herrick, 1985), are unlikely to retreat far enough to avoid exposure and by remaining *in situ* they might be exposed for long periods of time. Paralysis and mortalities have been observed in amphipods (*Eohaustorius estruarius*) residing up to 350 m from the edge of a salmonid net pen being treated with deltamethrin (Ernst et al., 2014; Lyons et al., 2017).

Aquatic invertebrates have shown pyrethroid LC50s less than 1 μg/L (Rehman et al., 2014). The larvae of the marine stone crab *Menippe mercenaria* have displayed a 96-h LC50 of 0.018 μg permethrin/L (Canadian Council of Ministers of the Environment, 2006). Permethrin has been shown to be significantly lethal to adult lobsters. Indeed, *H. americanus* specimens exposed to various permethrin concentrations have reached lethal thresholds between 0.40 and 0.68 μg/L as well as a 96-h LC50 of 0.73 μg/L (Zitko et al., 1979; McLeese et al., 1980). In this work, all stage IV American lobsters exposed to the formulation product containing permethrin at 100 μg/kg survived but had a significantly delayed growth (longer IP_45_ and decreased MI) compared to those exposed at 3 μg/kg and control lobsters. Sublethal effects (increased intermolt period and decreased SGR) were observed also in lobsters exposed to 3–10 μg/kg permethrin. Indeed, within 14 days of exposure, it was observed that only 40% of lobsters successfully molted to stage VI after being exposed to 3 μg/kg, compared to 60% of the control lobsters. In California, permethrin has been detected in coastal sediments at concentrations between 5 and 150 μg/kg, concentrations significantly higher than those used in this study (Méjanelle et al., 2020). Similarly to permethrin, another pyrethroid, fenvalerate, did not cause mortality but produced negative sublethal effects on sediment-exposed larvae and postlarvae of an estuarine shrimp, *Palaemonetes pugio* (McKenney et al., 1998). Larval metamorphosis and weight were inhibited when shrimp were exposed to sediment containing 100 μg/kg of fenvalerate. Moreover, the carbon and nitrogen content as well as the total energy reserves of the shrimp started being affected in larvae exposed to concentrations of 10 μg/kg (McKenney et al., 1998).

In contrast to the permethrin formulation, the deltamethrin product caused a dose-dependent increase in mortality, determining an LC50 of 2.5 µg active ingredient/kg. In another paper testing the effect of deltamethrin on the amphipod *Echinogammarus finmarchicus*, the 10-day LC50 was determined at 16 (CI 14–19) µg/kg in sediment (Van Geest et al., 2014). The 14-day LC50 of 2.5 μg/kg in this study is significantly lower than the above LC50s indicating that lobsters have a higher sensitivity to deltamethrin in comparison to *E*. *finmarchicus*.

Literature on the exposure of lobsters to pesticides is still scarce. Pesticides used in water bath treatment for sea lice control in salmon farms (Salmosan®, active ingredient: azamethiphos) caused acetylcholinesterase alterations followed by immobilization and mortality in American lobster larvae (Stage I-IV) in short-term exposures (de Jourdan et al., 2022). Deltamethrin exposure has been observed to reduce activity in shrimp (*Penaeus monodon*) and mortality and increased morbidity in stage I and II *H. gammarus*, in a dose-dependent manner (Tu et al., 2012; Parsons et al., 2020). Other studies showed that recovery from single pulse deltamethrin exposures did not typically occur in crustaceans, including stage I and II European lobsters (*H. gammarus*) and amphipods (*E. finmarchicus*), but rather the organisms displayed delayed toxicity, either immobilization or mortality after the single pulse exposures (Van Geest et al., 2014; Parsons et al., 2020). The difficulty of the European lobster in recovering from deltamethrin exposure provides further concern for exposed lobsters in the wild. Even if a wild lobster survives the initial exposure, it may not survive the longer-term effects of the pesticide. Combined with growth delays, reduced activity and moribundity cause a significant increase in the risk of predation. On the other hand, in the present study, in both experiments involving the deltamethrin-formulated product, no statistically significant differences emerged in the growth endpoints analysed in the organisms that survived and molted (SI, IP_45_, and SGR). This means that even at the highest concentration tested, the surviving juvenile lobsters’ features were not different from the control. However, a higher deformity occurrence was observed (Figure 4).

In the present study, the experimental design did not take into account precise observations of the lobsters’ appendages and the deformities herein observed were general. An increase in the occurrence of deformities however was observed with increased treatment concentration, reaching a peak of five out of seven lobsters affected in the 100 μg/kg permethrin exposed group. It was noted that European lobster, *H. gammarus*, exposed to the pyrethroid teflubenzuron and monitored for 3 months had an occurrence of deformities varying from 0% to 15% (Samuelsen et al., 2014). It is suspected that these deformities would affect feeding behaviour, respiration, and movements which may explain the delayed growth with increased treatment concentrations (Samuelsen et al., 2014). Future studies should involve more sublethal endpoints as behavioural observations, total energy balance determination, as well as biochemical and molecular biomarkers to understand the mode of action of these compounds.

Pollutant-mediated alterations to pathways involved in regulating cellular energy homeostasis can lead to a reduction in net energy balance, thereby reducing the survival potential, growth, development, and reproduction of an organism (Baird et al., 1990; Depledge et al., 1995; Fairchild et al., 1999; Hartwell, 2011). Measuring the impact of energy balance has been a problem that may be overcome by employing integrative approaches such as the scope for growth, metabolic scope, and cellular energy allocation, instead of focusing on a single bioenergetic parameter (Depledge et al., 1995; De Coen and Janssen, 1997). However, studies concerning the effects of pollutants on juvenile lobsters assessed mainly mortality or immobility. The present work is among the first that consider the effects of chemicals on the metabolic rate and metabolic scope of American lobster.

Early juvenile lobsters (stage IV) exposed to an acute sublethal dose of the organochlorine pesticide endosulfan showed no differences in growth, survival, or histological changes in the hepatopancreas (Daoud et al., 2014). Metabolic scope however was significantly decreased compared to control lobsters, which the authors suggest could have consequences for survival by impairing their ability to find food, shelter, or evade predators (Daoud et al., 2014). These observations have important implications for extrapolating effects observed on an individual level to higher levels of biological integration (i.e., population and hence lobster production).

Deltamethrin has been found to alter metabolism in aquatic organisms (Lu et al., 2019). For example, aerobic capacity was suppressed in deltamethrin exposed red swamp crayfish (*Procambarus clarkii*) (Wu et al., 2012). Hypoxia and metabolic stress in *P. clarkii* were correlated with decreased lactate dehydrogenase and increased lactic acid levels (Wu et al., 2012). In the present study, no significant differences were detected in the metabolic endpoints analysed on lobsters exposed to 0 and 0.5 μg/kg of deltamethrin. Nonetheless, while the reduction in FMS was not statistically significant (likely owing to a small and unequal sample size), it does point to a possible sublethal effect that could be explored in future studies.

It is worth pointing out that here the effects of two commercially available formulations have been assessed. The effect of active ingredients present in pesticides formulations may be changed by other compounds present in the solution or by environmental conditions (Pereira et al., 2009; Nagy et al., 2020; Asnicar et al., 2022), therefore new experiments should be carried out with the active ingredient alone. The hazard posed by pyrethroids based solely on experiments carried out either with only the active ingredient or the formulation may lead to inadequate conclusions (Nagy et al., 2020).

In conclusion, the current study showed that there are significant effects on the growth, molting success, and metabolism of juvenile American lobsters with differences between the two formulations tested. Permethrin’s main effects were sublethal, whereas deltamethrin increased the mortality of juvenile lobsters. Both compounds cause an increase in the occurrence of malformations. These effects could be detrimental to the survival of lobsters at this stage of development. Stage IV lobsters are integral to maintaining lobster populations and any decrease in survival at this stage may be reflected over time in the overall population in an area. Further research that should be pursued includes exposure to other stages of lobster development as well as an assessment of the impact of human activities on coastal environments. These activities may change and increase with global climate change and further studies are required to fully understand how these impacts will affect the American lobster in the future.

## Data Availability

The original contributions presented in the study are included in the article/Supplementary Material, further inquiries can be directed to the corresponding author.
