# Supplementary material for: Development, growth and metabolic effects in stage IV lobster (Homarus americanus) following chronic exposure to sediments spiked with commercial formulations of deltamethrin and permethrin
Source: Front Physiol. 2023 May 5;14:1151176. doi: 10.3389/fphys.2023.1151176 (PMC10198617; doi:10.3389/fphys.2023.1151176)
Supplement: Supplementary file 1 [file DataSheet1.docx]

Supplemental Information

**Figure S1.** Adapted from Daoud et al., 2007. “Temperature induced variation in oxygen consumption of juvenile and adult stages of the northern shrimp, *Pandalus borealis*.” https://doi-org.uml.idm.oclc.org/10.1016/j.jembe.2007.02.013


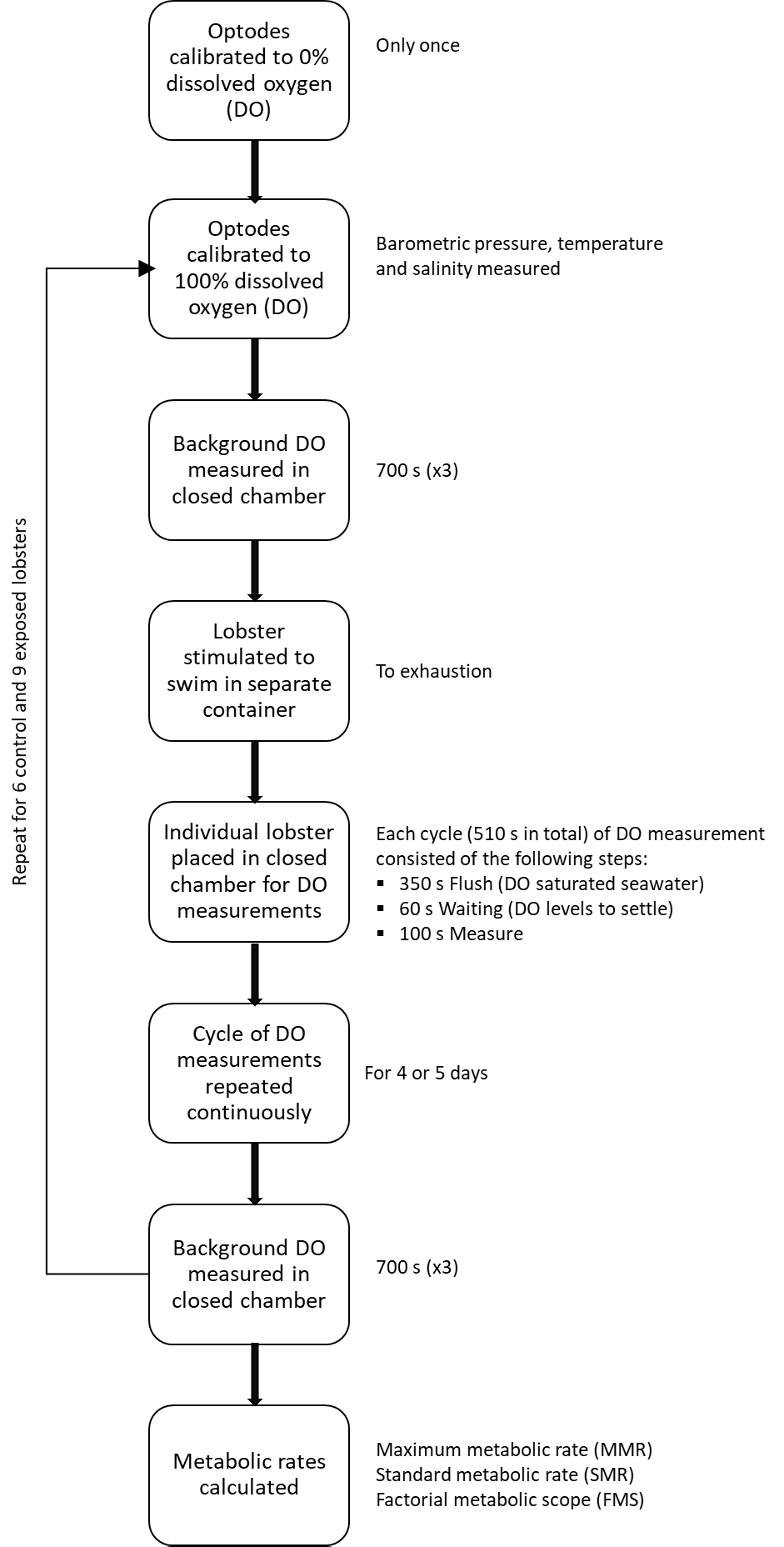


**Figure S2.** Flow chart of the Metabolic rate analysis.

Raw Data Table

| Year | Pesticide | Treatment Level | Nominal Concentration (ug/kg) | Lobster ID | Overall Size Increment (SI) | Intermolt Period 4 to 5 (IP45) | Specific Growth Rate Percent |
| --- | --- | --- | --- | --- | --- | --- | --- |
| 2012 | Permethrin (Trial 1) | 1 | 0 | HOM2012-266 | 30.47 | 12 | 0.96 |
|  |  |  |  | HOM2012-236 |  | 12 |  |
|  |  |  |  | HOM2012-327 | 13.16 | 12 | 0.45 |
|  |  |  |  | HOM2012-273 | 24.01 | 15 |  |
|  |  |  |  | HOM2012-326 | 48.55 | 12 | 0.82 |
|  |  |  |  | HOM2012-267 | 54.91 | 11 | 0.91 |
|  |  |  |  | HOM2012-237 |  | 12 |  |
|  |  |  |  | HOM2012-268 | 29.71 | 11 | 1.03 |
|  |  |  |  | HOM2012-239 |  | 12 |  |
|  |  |  |  | HOM2012-325 | 40.53 | 11 | 0.70 |
|  |  |  |  | HOM2012-320 | 43.29 | 11 | 0.74 |
|  |  |  |  | HOM2012-319 | 13.70 | 16 |  |
|  |  |  |  | HOM2012-274 | 29.27 | 12 | 0.93 |
|  |  |  |  | HOM2012-329 | 51.13 | 12 | 0.85 |
|  |  |  |  | HOM2012-328 | 8.62 | 17 |  |
|  |  |  |  | HOM2012-238 |  | 11 |  |
|  |  |  |  | HOM2012-275 | 38.91 | 12 | 1.19 |
|  |  |  |  | HOM2012-330 | 51.77 | 11 | 0.86 |
|  |  |  |  | HOM2012-269 | 34.98 | 12 | 1.09 |
|  |  |  |  | HOM2012-271 | 23.53 | 12 | 0.76 |
|  |  |  |  | HOM2012-270 | 30.09 | 11 | 1.04 |
|  |  |  |  | HOM2012-323 | 16.52 | 12 | 0.55 |
|  |  |  |  | HOM2012-240 |  | 12 |  |
|  |  |  |  | HOM2012-318 | 23.25 | 17 |  |
|  |  |  |  | HOM2012-316 | 50.08 | 12 | 0.84 |
|  |  |  |  | HOM2012-322 | 43.55 | 12 | 0.75 |
|  |  |  |  | HOM2012-241 |  | 11 |  |
|  |  |  |  | HOM2012-321 | 10.13 | 12 | 0.35 |
|  |  |  |  | HOM2012-272 | 26.25 | 12 | 0.84 |
|  |  |  |  | HOM2012-317 | 45.27 | 15 | 0.77 |
|  |  |  |  | HOM2012-324 | 48.16 | 11 | 0.81 |
|  |  | 2 | 3 | HOM2012-341 | 35.63 | 11 | 1.20 |
|  |  |  |  | HOM2012-242 |  | 12 |  |
|  |  |  |  | HOM2012-338 | 52.83 | 11 | 0.88 |
|  |  |  |  | HOM2012-276 | 26.07 | 12 | 0.48 |
|  |  |  |  | HOM2012-335 | 47.39 | 11 | 0.80 |
|  |  |  |  | HOM2012-243 |  | 12 |  |
|  |  |  |  | HOM2012-337 | 45.60 | 12 | 0.78 |
|  |  |  |  | HOM2012-339 | 32.45 | 11 | 1.11 |
|  |  |  |  | HOM2012-244 |  | 12 |  |
|  |  |  |  | HOM2012-277 | 42.30 | 12 | 0.73 |
|  |  |  |  | HOM2012-340 | 34.20 | 12 | 1.06 |
|  |  |  |  | HOM2012-245 |  | 12 |  |
|  |  |  |  | HOM2012-279 | 24.81 | 12 | 0.80 |
|  |  |  |  | HOM2012-246 |  | 16 |  |
|  |  |  |  | HOM2012-284 | 12.19 | 16 | 0.31 |
|  |  |  |  | HOM2012-342 | 51.80 | 12 | 0.86 |
|  |  |  |  | HOM2012-331 | 54.41 | 12 | 0.90 |
|  |  |  |  | HOM2012-336 | 42.29 | 12 | 0.73 |
|  |  |  |  | HOM2012-333 | 15.70 | 12 | 0.53 |
|  |  |  |  | HOM2012-334 | 30.40 | 12 | 0.96 |
|  |  |  |  | HOM2012-278 | 47.51 | 11 | 0.84 |
|  |  |  |  | HOM2012-280 | 13.62 | 12 | 0.46 |
|  |  |  |  | HOM2012-332 | 12.44 | 12 | 0.27 |
|  |  |  |  | HOM2012-281 | 23.51 | 17 | 0.54 |
|  |  |  |  | HOM2012-247 |  | 13 |  |
|  |  |  |  | HOM2012-282 | 28.51 | 16 | 0.68 |
|  |  |  |  | HOM2012-344 | 46.32 | 12 | 0.79 |
|  |  |  |  | HOM2012-343 | 56.69 | 12 | 0.93 |
|  |  |  |  | HOM2012-345 | 49.55 | 12 | 0.83 |
|  |  |  |  | HOM2012-285 | 35.17 | 12 | 1.09 |
|  |  |  |  | HOM2012-283 | 55.66 | 12 | 0.92 |
|  |  | 3 | 10 | HOM2012-351 | 35.86 | 16 | 0.63 |
|  |  |  |  | HOM2012-348 | 62.84 | 12 | 1.01 |
|  |  |  |  | HOM2012-248 |  | 12 |  |
|  |  |  |  | HOM2012-286 | 28.07 | 12 | 0.90 |
|  |  |  |  | HOM2012-358 | 3.68 | 11 | 0.14 |
|  |  |  |  | HOM2012-287 | 36.84 | 17 | 0.80 |
|  |  |  |  | HOM2012-347 |  | 12 |  |
|  |  |  |  | HOM2012-288 | 16.80 | 12 | 0.32 |
|  |  |  |  | HOM2012-253 |  | 12 |  |
|  |  |  |  | HOM2012-292 | 20.13 | 15 | 0.53 |
|  |  |  |  | HOM2012-354 | 12.59 | 19 | 0.27 |
|  |  |  |  | HOM2012-289 | 32.76 | 19 | 0.65 |
|  |  |  |  | HOM2012-349 | 17.75 | 19 | 0.37 |
|  |  |  |  | HOM2012-346 | 16.49 | 12 | 0.55 |
|  |  |  |  | HOM2012-352 | 15.29 | 12 | 0.51 |
|  |  |  |  | HOM2012-290 | 24.30 | 20 | 0.47 |
|  |  |  |  | HOM2012-350 | 17.26 | 12 | 0.58 |
|  |  |  |  | HOM2012-291 | 25.94 | 17 | 0.59 |
|  |  |  |  | HOM2012-252 |  | 12 |  |
|  |  |  |  | HOM2012-357 | 56.31 | 12 | 0.92 |
|  |  |  |  | HOM2012-251 |  | 12 |  |
|  |  |  |  | HOM2012-353 | 15.64 | 12 | 0.53 |
|  |  |  |  | HOM2012-294 | 22.10 | 12 | 0.72 |
|  |  |  |  | HOM2012-360 | 39.42 | 12 | 0.69 |
|  |  |  |  | HOM2012-250 |  | 12 |  |
|  |  |  |  | HOM2012-293 | 21.03 | 17 | 0.49 |
|  |  |  |  | HOM2012-295 | 47.75 | 11 | 0.81 |
|  |  |  |  | HOM2012-355 | 16.85 | 20 | 0.34 |
|  |  |  |  | HOM2012-249 |  | 12 |  |
|  |  |  |  | HOM2012-359 | 26.05 | 19 | 0.53 |
|  |  |  |  | HOM2012-356 | 52.42 | 12 | 0.87 |
|  |  | 4 | 33 | HOM2012-296 | 66.55 | 12 | 1.05 |
|  |  |  |  | HOM2012-366 | 11.08 | 12 | 0.38 |
|  |  |  |  | HOM2012-361 | 18.80 | 12 | 0.36 |
|  |  |  |  | HOM2012-297 | 24.32 | 12 | 0.79 |
|  |  |  |  | HOM2012-362 | 19.05 | 19 | 0.40 |
|  |  |  |  | HOM2012-303 | 30.46 | 12 | 0.96 |
|  |  |  |  | HOM2012-259 |  | 12 |  |
|  |  |  |  | HOM2012-365 | 39.14 | 12 | 0.68 |
|  |  |  |  | HOM2012-363 | 56.36 | 11 | 0.92 |
|  |  |  |  | HOM2012-364 | 26.76 | 15 | 0.69 |
|  |  |  |  | HOM2012-304 | 35.81 | 12 | 0.63 |
|  |  |  |  | HOM2012-371 | 18.85 | 12 | 0.62 |
|  |  |  |  | HOM2012-298 | 38.31 | 12 | 1.17 |
|  |  |  |  | HOM2012-305 | 34.38 | 16 | 0.80 |
|  |  |  |  | HOM2012-258 |  | 12 |  |
|  |  |  |  | HOM2012-367 | 20.87 | 18 | 0.46 |
|  |  |  |  | HOM2012-370 | 40.50 | 11 | 0.70 |
|  |  |  |  | HOM2012-372 | 24.25 | 15 | 0.63 |
|  |  |  |  | HOM2012-257 |  | 14 |  |
|  |  |  |  | HOM2012-299 | 27.96 | 15 | 0.71 |
|  |  |  |  | HOM2012-368 | 24.36 | 13 | 0.73 |
|  |  |  |  | HOM2012-300 | 28.73 | 12 | 0.91 |
|  |  |  |  | HOM2012-256 |  | 14 |  |
|  |  |  |  | HOM2012-373 | 23.89 | 18 | 0.52 |
|  |  |  |  | HOM2012-302 | 22.87 | 12 | 0.75 |
|  |  |  |  | HOM2012-255 |  | 14 |  |
|  |  |  |  | HOM2012-301 | 32.58 | 12 | 1.02 |
|  |  |  |  | HOM2012-409 |  |  |  |
|  |  |  |  | HOM2012-254 |  | 12 |  |
|  |  |  |  | HOM2012-410 |  | 12 |  |
|  |  |  |  | HOM2012-369 | 18.31 | 12 | 0.61 |
|  |  | 5 | 100 | HOM2012-260 |  | 19 |  |
|  |  |  |  | HOM2012-378 | 17.64 | 19 | 0.37 |
|  |  |  |  | HOM2012-306 | 16.77 | 19 | 0.35 |
|  |  |  |  | HOM2012-261 |  | 19 |  |
|  |  |  |  | HOM2012-374 | 44.80 | 15 | 0.77 |
|  |  |  |  | HOM2012-262 |  | 13 |  |
|  |  |  |  | HOM2012-307 | 16.75 | 19 | 0.35 |
|  |  |  |  | HOM2012-315 | 22.64 | 16 | 0.55 |
|  |  |  |  | HOM2012-263 |  | 15 |  |
|  |  |  |  | HOM2012-375 | 8.70 | 18 | 0.20 |
|  |  |  |  | HOM2012-308 | 32.01 | 15 | 0.80 |
|  |  |  |  | HOM2012-314 | 27.22 | 12 | 0.87 |
|  |  |  |  | HOM2012-264 |  | 19 |  |
|  |  |  |  | HOM2012-379 | 19.13 | 19 | 0.40 |
|  |  |  |  | HOM2012-313 | 18.22 | 19 | 0.38 |
|  |  |  |  | HOM2012-381 | 0.24 | 19 | 0.01 |
|  |  |  |  | HOM2012-384 | 18.41 | 16 | 0.46 |
|  |  |  |  | HOM2012-265 |  | 13 |  |
|  |  |  |  | HOM2012-388 | 48.37 | 17 | 1.01 |
|  |  |  |  | HOM2012-387 | 53.93 | 12 | 0.89 |
|  |  |  |  | HOM2012-309 | 19.43 | 13 | 0.59 |
|  |  |  |  | HOM2012-383 | 7.62 | 16 | 0.20 |
|  |  |  |  | HOM2012-310 | 20.42 | 12 | 0.67 |
|  |  |  |  | HOM2012-380 | 17.41 | 19 | 0.37 |
|  |  |  |  | HOM2012-376 | 30.43 | 18 | 0.64 |
|  |  |  |  | HOM2012-311 | 16.40 | 20 | 0.33 |
|  |  |  |  | HOM2012-386 | 19.08 | 19 | 0.40 |
|  |  |  |  | HOM2012-312 | 26.53 | 19 | 0.54 |
|  |  |  |  | HOM2012-385 | 15.98 | 19 | 0.34 |
|  |  |  |  | HOM2012-382 | 16.36 | 18 | 0.37 |
|  |  |  |  | HOM2012-377 | 15.49 | 19 | 0.33 |
| 2013 | Deltamethrin (Trial 2) | 1 | 0 | HOM2013-34 |  |  |  |
|  |  |  |  | HOM2013-35 |  |  |  |
|  |  |  |  | HOM2013-36 |  |  |  |
|  |  |  |  | HOM2013-37 |  |  |  |
|  |  |  |  | HOM2013-38 |  |  |  |
|  |  |  |  | HOM2013-52 |  | 14 |  |
|  |  |  |  | HOM2013-53 |  | 12 |  |
|  |  |  |  | HOM2013-54 |  | 14 |  |
|  |  |  |  | HOM2013-55 |  | 10 |  |
|  |  |  |  | HOM2013-56 |  | 11 |  |
|  |  |  |  | HOM2013-57 |  | 12 |  |
|  |  |  |  | HOM2013-76 | 13.67 | 16 | 0.35 |
|  |  |  |  | HOM2013-77 |  | 16 |  |
|  |  |  |  | HOM2013-78 | 7.67 | 14 | 0.23 |
|  |  |  |  | HOM2013-79 | 15.59 | 12 | 0.52 |
|  |  |  |  | HOM2013-80 | 36.69 | 11 | 1.23 |
|  |  |  |  | HOM2013-81 | 9.35 | 10 | 0.39 |
|  |  |  |  | HOM2013-82 | 4.56 | 12 | 0.16 |
|  |  |  |  | HOM2013-83 | 11.27 | 16 | 0.29 |
|  |  |  |  | HOM2013-84 | 30.94 | 11 | 1.06 |
|  |  |  |  | HOM2013-85 | 20.14 | 13 | 0.61 |
|  |  |  |  | HOM2013-86 | 16.55 | 12 | 0.55 |
|  |  |  |  | HOM2013-87 | 14.63 | 12 | 0.49 |
|  |  |  |  | HOM2013-88 | 15.35 | 14 | 0.44 |
|  |  |  |  | HOM2013-101 | 15.83 | 13 | 0.49 |
|  |  |  |  | HOM2013-102 | 14.39 | 14 | 0.42 |
|  |  |  |  | HOM2013-103 | 4.56 | 11 | 0.18 |
|  |  |  |  | HOM2013-104 | 6.00 | 16 | 0.16 |
|  |  |  |  | HOM2013-105 | 19.42 | 16 | 0.48 |
|  |  |  |  | HOM2013-106 | 9.11 | 14 | 0.27 |
|  |  |  |  | HOM2013-107 | 16.79 | 12 | 0.56 |
|  |  |  |  | HOM2013-108 | 6.95 | 14 | 0.21 |
|  |  |  |  | HOM2013-109 | 10.55 | 15 | 0.29 |
|  |  |  |  | HOM2013-110 | 1.68 | 16 | 0.05 |
|  |  |  |  | HOM2013-111 | 9.11 | 14 | 0.27 |
|  |  | 2 | 0.05 | HOM2013-39 |  |  |  |
|  |  |  |  | HOM2013-40 |  |  |  |
|  |  |  |  | HOM2013-41 |  |  |  |
|  |  |  |  | HOM2013-42 |  |  |  |
|  |  |  |  | HOM2013-43 |  |  |  |
|  |  |  |  | HOM2013-58 |  | 16 |  |
|  |  |  |  | HOM2013-59 |  | 13 |  |
|  |  |  |  | HOM2013-60 |  | 14 |  |
|  |  |  |  | HOM2013-61 |  | 11 |  |
|  |  |  |  | HOM2013-62 |  | 12 |  |
|  |  |  |  | HOM2013-63 |  | 10 |  |
|  |  |  |  | HOM2013-89 | 20.38 | 16 | 0.50 |
|  |  |  |  | HOM2013-90 | 1.68 | 14 | 0.05 |
|  |  |  |  | HOM2013-91 | 15.11 | 13 | 0.47 |
|  |  |  |  | HOM2013-92 | 17.75 | 11 | 0.64 |
|  |  |  |  | HOM2013-93 | 11.99 | 12 | 0.41 |
|  |  |  |  | HOM2013-94 | 11.99 | 12 | 0.41 |
|  |  |  |  | HOM2013-112 | 9.59 | 16 | 0.25 |
|  |  |  |  | HOM2013-113 | 20.38 | 12 | 0.67 |
|  |  |  |  | HOM2013-114 | 4.80 | 16 | 0.13 |
|  |  |  |  | HOM2013-115 | 18.71 | 12 | 0.62 |
|  |  |  |  | HOM2013-116 | 14.63 | 13 | 0.46 |
|  |  |  |  | HOM2013-117 | 10.31 | 12 | 0.36 |
|  |  |  |  | HOM2013-118 |  |  |  |
|  |  |  |  | HOM2013-119 | 19.42 | 13 | 0.59 |
|  |  |  |  | HOM2013-120 |  | 13 |  |
|  |  |  |  | HOM2013-121 | 10.79 | 13 | 0.34 |
|  |  |  |  | HOM2013-122 | 5.04 | 11 | 0.19 |
|  |  |  |  | HOM2013-123 | 13.19 | 13 | 0.41 |
|  |  |  |  | HOM2013-124 | 13.19 | 12 | 0.45 |
|  |  |  |  | HOM2013-125 |  | 14 |  |
|  |  |  |  | HOM2013-126 | 17.99 | 15 | 0.48 |
|  |  |  |  | HOM2013-127 | 8.15 | 14 | 0.24 |
|  |  |  |  | HOM2013-128 | 7.67 | 13 | 0.25 |
|  |  |  |  | HOM2013-129 | 6.47 | 13 | 0.21 |
|  |  |  |  | HOM2013-130 | 3.60 | 12 | 0.13 |
|  |  | 3 | 0.5 | HOM2013-44 |  |  |  |
|  |  |  |  | HOM2013-45 |  |  |  |
|  |  |  |  | HOM2013-46 |  |  |  |
|  |  |  |  | HOM2013-47 |  |  |  |
|  |  |  |  | HOM2013-48 |  |  |  |
|  |  |  |  | HOM2013-64 |  | 10 |  |
|  |  |  |  | HOM2013-65 |  | 12 |  |
|  |  |  |  | HOM2013-66 |  | 14 |  |
|  |  |  |  | HOM2013-67 |  | 13 |  |
|  |  |  |  | HOM2013-68 |  | 11 |  |
|  |  |  |  | HOM2013-69 |  | 14 |  |
|  |  |  |  | HOM2013-95 | 6.00 | 12 | 0.21 |
|  |  |  |  | HOM2013-96 | 11.51 | 13 | 0.36 |
|  |  |  |  | HOM2013-97 | 6.95 | 11 | 0.27 |
|  |  |  |  | HOM2013-98 | 6.47 | 16 | 0.17 |
|  |  |  |  | HOM2013-99 | 10.31 | 15 | 0.28 |
|  |  |  |  | HOM2013-100 | 11.03 | 14 | 0.32 |
|  |  |  |  | HOM2013-131 | 12.23 | 12 | 0.42 |
|  |  |  |  | HOM2013-132 | 11.99 | 12 | 0.41 |
|  |  |  |  | HOM2013-133 | 13.91 | 13 | 0.44 |
|  |  |  |  | HOM2013-134 | 23.02 | 11 | 0.82 |
|  |  |  |  | HOM2013-135 | 22.78 | 11 | 0.81 |
|  |  |  |  | HOM2013-136 | 7.19 | 16 | 0.19 |
|  |  |  |  | HOM2013-137 | 8.39 | 11 | 0.32 |
|  |  |  |  | HOM2013-138 | 9.83 | 12 | 0.34 |
|  |  |  |  | HOM2013-139 | 8.39 | 11 | 0.32 |
|  |  |  |  | HOM2013-140 | 4.56 | 13 | 0.15 |
|  |  |  |  | HOM2013-141 | 9.35 | 15 | 0.26 |
|  |  |  |  | HOM2013-142 | 4.80 | 11 | 0.18 |
|  |  |  |  | HOM2013-143 | 6.71 |  |  |
|  |  |  |  | HOM2013-144 |  | 13 |  |
|  |  |  |  | HOM2013-145 | 12.71 | 11 | 0.47 |
|  |  |  |  | HOM2013-146 | 26.86 | 14 | 0.74 |
|  |  |  |  | HOM2013-147 | 8.15 | 13 | 0.26 |
|  |  |  |  | HOM2013-148 | 20.38 | 13 | 0.62 |
|  |  | 4 | 5 | HOM2013-49 |  |  |  |
|  |  |  |  | HOM2013-50 |  |  |  |
|  |  |  |  | HOM2013-51 |  |  |  |
|  |  |  |  | HOM2013-70 |  | 12 |  |
|  |  |  |  | HOM2013-71 |  | 15 |  |
|  |  |  |  | HOM2013-72 |  | 16 |  |
|  |  |  |  | HOM2013-73 |  |  |  |
|  |  |  |  | HOM2013-74 |  | 14 |  |
|  |  |  |  | HOM2013-75 |  | 14 |  |
|  | Deltamethrin (Trial 3) | 1 | 0 | HOM2013-179 |  |  |  |
|  |  |  |  | HOM2013-180 | 1.67 |  |  |
|  |  |  |  | HOM2013-181 | 9.07 |  |  |
|  |  |  |  | HOM2013-182 |  |  |  |
|  |  |  |  | HOM2013-183 | 1.19 |  |  |
|  |  |  |  | HOM2013-189 | 16.71 | 9 | 0.75 |
|  |  |  |  | HOM2013-190 | 8.35 | 10 | 0.35 |
|  |  |  |  | HOM2013-191 | 21.72 | 11 | 0.78 |
|  |  |  |  | HOM2013-192 | 15.51 | 12 | 0.52 |
|  |  |  |  | HOM2013-193 | 4.53 | 11 | 0.18 |
|  |  |  |  | HOM2013-194 | 5.73 | 10 | 0.24 |
|  |  |  |  | HOM2013-195 | 20.29 | 11 | 0.73 |
|  |  |  |  | HOM2013-196 | 3.34 | 11 | 0.13 |
|  |  |  |  | HOM2013-197 | 10.02 | 12 | 0.35 |
|  |  |  |  | HOM2013-198 | 17.90 | 10 | 0.72 |
|  |  |  |  | HOM2013-199 | 23.39 | 11 | 0.83 |
|  |  |  |  | HOM2013-200 | 11.46 | 11 | 0.43 |
|  |  |  |  | HOM2013-201 | 15.99 | 12 | 0.54 |
|  |  |  |  | HOM2013-202 | 1.43 | 12 | 0.05 |
|  |  |  |  | HOM2013-203 | 6.44 | 12 | 0.23 |
|  |  |  |  | HOM2013-204 | 8.83 | 10 | 0.37 |
|  |  |  |  | HOM2013-205 | 10.26 | 13 | 0.33 |
|  |  |  |  | HOM2013-206 | 15.51 | 11 | 0.57 |
|  |  |  |  | HOM2013-207 | 11.69 | 10 | 0.48 |
|  |  |  |  | HOM2013-208 | 18.38 | 9 | 0.81 |
|  |  |  |  | HOM2013-229 | 13.37 | 12 | 0.45 |
|  |  |  |  | HOM2013-230 | 9.79 | 12 | 0.34 |
|  |  |  |  | HOM2013-231 | 13.60 | 11 | 0.50 |
|  |  |  |  | HOM2013-232 | 19.81 | 13 | 0.60 |
|  |  |  |  | HOM2013-233 | 18.85 | 10 | 0.75 |
|  |  |  |  | HOM2013-234 | 5.01 | 13 | 0.16 |
|  |  |  |  | HOM2013-235 | 17.18 | 11 | 0.63 |
|  |  |  |  | HOM2013-236 | 18.85 | 9 | 0.83 |
|  |  |  |  | HOM2013-237 | 10.02 | 11 | 0.38 |
|  |  |  |  | HOM2013-247 | 21.72 | 11 | 0.78 |
|  |  |  |  | HOM2013-248 | 14.08 | 12 | 0.48 |
|  |  |  |  | HOM2013-249 | 12.89 | 10 | 0.53 |
|  |  |  |  | HOM2013-250 | 5.73 | 11 | 0.22 |
|  |  |  |  | HOM2013-251 | 4.30 | 10 | 0.18 |
|  |  |  |  | HOM2013-252 | 24.11 | 13 | 0.72 |
|  |  |  |  | HOM2013-253 | 26.73 | 12 | 0.86 |
|  |  |  |  | HOM2013-254 | 29.12 | 9 | 1.23 |
|  |  |  |  | HOM2013-255 | 14.08 | 13 | 0.44 |
|  |  |  |  | HOM2013-256 | 32.22 | 10 | 1.21 |
|  |  |  |  | HOM2013-257 | 22.91 | 12 | 0.75 |
|  |  |  |  | HOM2013-258 | 21.00 | 10 | 0.83 |
|  |  |  |  | HOM2013-259 |  | 14 |  |
|  |  |  |  | HOM2013-260 | 17.18 | 11 | 0.63 |
|  |  |  |  | HOM2013-261 | 25.30 | 11 | 0.89 |
|  |  |  |  | HOM2013-262 | 14.32 | 11 | 0.53 |
|  |  |  |  | HOM2013-263 | 12.17 | 12 | 0.42 |
|  |  | 3 | 0.5 | HOM2013-184 | 5.25 |  |  |
|  |  |  |  | HOM2013-185 |  |  |  |
|  |  |  |  | HOM2013-186 | 3.34 |  |  |
|  |  |  |  | HOM2013-187 |  |  |  |
|  |  |  |  | HOM2013-188 |  |  |  |
|  |  |  |  | HOM2013-209 | 13.60 | 12 | 0.46 |
|  |  |  |  | HOM2013-210 | 14.56 | 13 | 0.45 |
|  |  |  |  | HOM2013-211 | 23.39 | 11 | 0.83 |
|  |  |  |  | HOM2013-212 | 9.79 | 12 | 0.34 |
|  |  |  |  | HOM2013-213 | 13.84 | 15 | 0.38 |
|  |  |  |  | HOM2013-214 | 19.81 | 11 | 0.71 |
|  |  |  |  | HOM2013-215 | 8.35 | 11 | 0.32 |
|  |  |  |  | HOM2013-216 | 8.11 | 13 | 0.26 |
|  |  |  |  | HOM2013-217 | 9.07 | 12 | 0.31 |
|  |  |  |  | HOM2013-218 | 22.91 | 11 | 0.81 |
|  |  |  |  | HOM2013-219 | 16.95 | 14 | 0.49 |
|  |  |  |  | HOM2013-220 | 17.42 | 11 | 0.63 |
|  |  |  |  | HOM2013-221 | 14.32 | 12 | 0.48 |
|  |  |  |  | HOM2013-222 | 22.91 | 11 | 0.81 |
|  |  |  |  | HOM2013-223 | 20.53 | 10 | 0.81 |
|  |  |  |  | HOM2013-224 | 17.42 | 11 | 0.63 |
|  |  |  |  | HOM2013-225 | 28.40 | 9 | 1.21 |
|  |  |  |  | HOM2013-226 | 14.80 | 11 | 0.54 |
|  |  |  |  | HOM2013-227 | 9.79 | 10 | 0.41 |
|  |  |  |  | HOM2013-228 | 14.32 | 10 | 0.58 |
|  |  |  |  | HOM2013-238 | 28.40 | 10 | 1.09 |
|  |  |  |  | HOM2013-239 | 18.38 | 11 | 0.67 |
|  |  |  |  | HOM2013-240 | 17.42 | 12 | 0.58 |
|  |  |  |  | HOM2013-241 | 13.13 | 12 | 0.45 |
|  |  |  |  | HOM2013-242 | 12.89 | 10 | 0.53 |
|  |  |  |  | HOM2013-243 | 13.37 | 11 | 0.50 |
|  |  |  |  | HOM2013-244 | 18.62 | 11 | 0.67 |
|  |  |  |  | HOM2013-245 | 20.53 | 10 | 0.81 |
|  |  |  |  | HOM2013-246 | 17.18 | 13 | 0.53 |
|  |  |  |  | HOM2013-264 | 21.96 | 11 | 0.78 |
|  |  |  |  | HOM2013-265 | 23.63 | 10 | 0.92 |
|  |  |  |  | HOM2013-266 | 12.41 | 12 | 0.42 |
|  |  |  |  | HOM2013-267 | 16.71 | 11 | 0.61 |
|  |  |  |  | HOM2013-268 | 13.13 | 12 | 0.45 |
|  |  |  |  | HOM2013-269 | 13.13 | 14 | 0.38 |
|  |  |  |  | HOM2013-270 | 18.62 | 11 | 0.67 |
|  |  |  |  | HOM2013-271 | 15.04 | 12 | 0.51 |
|  |  |  |  | HOM2013-272 | 15.51 | 12 | 0.52 |
|  |  |  |  | HOM2013-273 | 34.84 | 10 | 1.30 |
|  |  |  |  | HOM2013-274 | 20.76 | 14 | 0.59 |
|  |  |  |  | HOM2013-275 | 18.38 | 11 | 0.67 |
|  |  |  |  | HOM2013-276 | 17.18 | 10 | 0.69 |
|  |  |  |  | HOM2013-277 | 13.37 | 10 | 0.54 |
|  |  |  |  | HOM2013-278 | 14.08 | 10 | 0.57 |
|  |  |  |  | HOM2013-279 | 58.47 | 13 | 1.54 |
|  |  |  |  | HOM2013-280 | 11.22 | 10 | 0.46 |

# Concentration Response Modelling

## Permethrin Concentration Response

Percent of lobsters which molted to stage VI was modelled with a three-parameter type 2 Weibull model


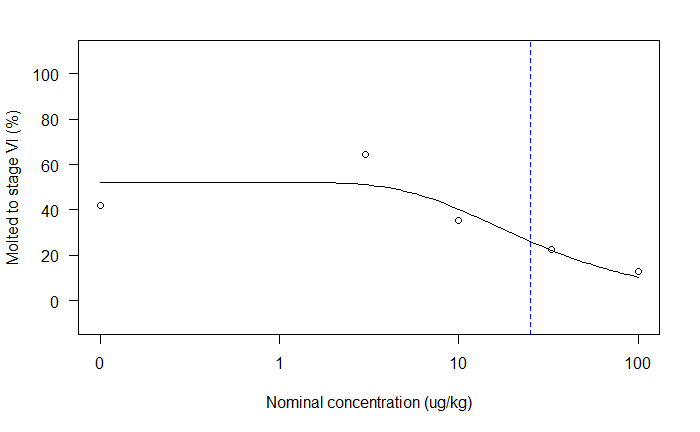


Model fitted: Weibull (type 2) with lower limit at 0 (3 parms)

Parameter estimates:

| Parameter | Estimate | Std. Error | t-value | p-value |
| --- | --- | --- | --- | --- |
| b | -0.82253 | 0.41727 | -1.9712 | 0.18748 |
| d | 52.11668 | 9.07808 | 5.7409 | 0.02903 |
| e | 16.05129 | 10.60473 | 1.5136 | 0.26931 |

Residual standard error:

12.48063 (2 degrees of freedom)

Estimated effective doses

Estimated Molting Effect Levels

| Effect Level (%) | Estimate (µg/kg) | Std. Error |
| --- | --- | --- |
| 20 | 9.0000 | 6.3218 |
| 50 | 25.0628 | 17.9004 |
| 90 | 247.5749 | 391.5392 |

## Deltamethrin Concentration Response

Cumulative mortality after 14-d exposure


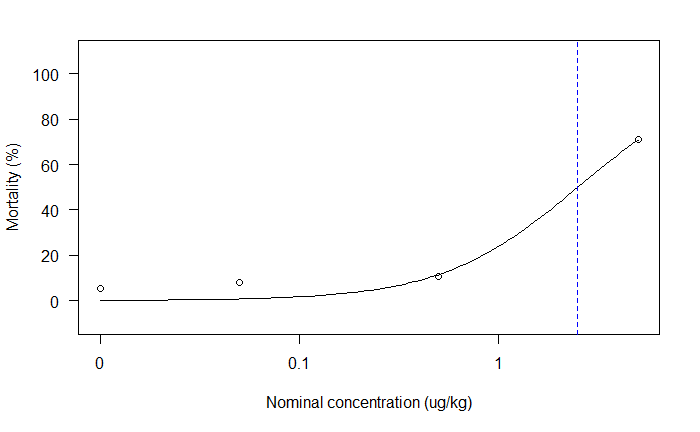


Model fitted: Log-logistic (LC50 as parameter) (2 parms, lower and upper limits fixed to 0 and 100)

Parameter estimates:

| Parameter | Estimate | Std. Error | t-value | p-value |
| --- | --- | --- | --- | --- |
| b | -1.27126 | 0.32189 | -3.9493 | 0.05854 |
| e | 2.49443 | 0.56342 | 4.4273 | 0.04742 |

Residual standard error:

6.367456 (2 degrees of freedom)

Estimated Lethal Effect Levels

| Effect Level (%) | Estimate (µg/kg) | Std. Error |
| --- | --- | --- |
| 20 | 0.83826 | 0.33873 |
| 50 | 2.49443 | 0.56342 |
| 90 | 14.04740 | 6.04932 |
